# Trends in insecticide resistance in *Culex pipiens pallens* over 20 years in Shandong, China

**DOI:** 10.1186/s13071-019-3416-9

**Published:** 2019-04-11

**Authors:** Hongmei Liu, Lihua Xie, Peng Cheng, Jiabao Xu, Xiaodan Huang, Haifang Wang, Xiao Song, Lijuan Liu, Huaiwei Wang, Jingxuan Kou, Guiyun Yan, Xiao-Guang Chen, Maoqing Gong

**Affiliations:** 1 Department of Medical Entomology, Shandong Academy of Medical Sciences, Shandong Institute of Parasitic Diseases, Jining, 272033 Shandong People’s Republic of China; 2 Department of Pathogen Biology, Guangdong Provincial Key Laboratory of Tropical Disease Research, School of Public Health, Southern Medical University, Guangzhou, People’s Republic of China; 3 Program in Public Health, University of California, Irvine, CA USA

**Keywords:** *Culex pipiens pallens*, Insecticide resistance, *kdr*, L1014F, L1014S

## Abstract

**Background:**

*Culex pipiens pallens* is the most abundant *Culex* mosquito species in northern China and is an important vector of bancroftian filariasis and, potentially, West Nile virus. Insecticides, particularly pyrethroids, are widely used for adult mosquito control. Insecticide resistance has become common in several mosquito species, and vector control is the main method currently available to prevent disease transmission. The voltage-gated sodium channel (*Vgsc*) gene is the target site of pyrethroids, and mutations in this gene cause knockdown resistance (*kdr*).

**Methods:**

*Culex pipiens pallens* larvae were collected from May to November over two decades, from 1992 to 2018, in four cities in Shandong Province, China. The World Health Organization (WHO) standard resistance bioassay was applied to test the resistance levels of *Cx. p. pallens* larvae to five different insecticides and to test deltamethrin resistance in adults, using the F1 generation. Mutations at *Vgsc* codon 1014 were also screened in 471 adult samples collected in 2014 to determine the association between *kdr* mutations and phenotypic resistance.

**Results:**

Larval resistance against deltamethrin showed an increasing trend from the 1990s until 2018, which was statistically significant in all populations; resistance to cypermethrin increased significantly in mosquitoes from the Zaozhuang population. However, larval resistance to other insecticides remained relatively stable. Larval resistance against deltamethrin was consistent with adult bioassays in 2014, in which all tested populations were highly resistant, with mortality rates ranging from 39.4 to 55.23%. The L1014S and L1014F mutations were both observed in five *Cx. p. pallens* populations, with L1014F significantly associated with deltamethrin resistance.

**Conclusions:**

The long-term dataset from Shandong demonstrates major increases in pyrethroid resistance over a 20-year period. The L1014F *kdr* mutation may be considered a viable molecular marker for monitoring pyrethroid resistance in *Cx. p. pallens.*

**Electronic supplementary material:**

The online version of this article (10.1186/s13071-019-3416-9) contains supplementary material, which is available to authorized users.

## Background

*Culex pipiens pallens* is widely distributed in China [1, 2]. This species usually occurs at a high density near humans and can transmit bancroftian filariasis and Japanese encephalitis. *Culex pipiens pallens* is also a potential vector of West Nile virus (WNV) in China [3]. The control of mosquito populations is mainly based on chemical insecticides. Insecticide-treated bednets (ITNs) and indoor residual spraying (IRS) are the primary vector control methods recommended by the WHO [4]. Currently, pyrethroids are the principal class of insecticide approved for use on ITNs and are the most widely employed insecticide for vector control programs due to their low mammalian toxicity and rapid knockdown action [5]. Pyrethroids, organochlorines, organophosphates and carbamates are the four main classes of insecticide approved for IRS [6]. Among the four synthetic insecticide families, deltamethrin and cypermethrin are pyrethroids, dichlorvos (DDVP) is an organophosphate, propoxur is a carbamate, and acetofenate is an organochlorine; all of these insecticide have been or are now widely used in China [2]. In India, the use of vector control insecticides was dominated by DDT and pyrethroids in the 2000s [7]. China has banned the application of some organochlorine pesticides by replacing them with carbamate and pyrethroid insecticides since 1983 [8]. Prolonged and frequent use of insecticides imposes selection pressure on mosquito populations and will usually lead to resistance, reducing efficacy of the class of insecticides and limiting the available options for mosquito control. Monitoring insecticide resistance is essential for effective management of resistance and is therefore important for the planning of control measures and disease vector control interventions to counteract various mosquito-borne diseases. However, there is a lack of systematic and continuous research on the resistance levels of *Cx. p. pallens* in China.

Pyrethroids target the *Vgsc* (voltage-gated sodium channel) gene of insect neurons. A major mechanism underlying pyrethroid resistance is reduced target site sensitivity resulting from non-synonymous mutations in the *Vgsc* gene, resulting from single amino-acid substitutions that have been shown to be correlated with phenotypic resistance to pyrethroids. This form of resistance, known as knockdown resistance (*kdr*), has been observed in a number of mosquitoes, including *Anopheles gambiae* [9, 10], *An. sinensis* [11–14], *Cx. quinquefasciatus* [3, 15, 16] and *Ae. aegypti* [17–19]. In *An. gambiae*, L1014F and L1014S in domain II of subunit 6 (IIS6) of the *Vgsc* gene are the best-studied mutations related to pyrethroid and DDT resistance [20–23].

In the present study, we collected *Cx. p. pallens* larvae from 1992 to 2018 to examine their resistance levels to the major insecticide families. An adult bioassay was also conducted in 2014 to examine pyrethroid resistance and the correlation of L1014F and L1014S *kdr* mutations with mosquito survivorship to provide insight into the role of *kdr* mutations in pyrethroid resistance.

## Methods

### Study areas

**Fig. 1:**
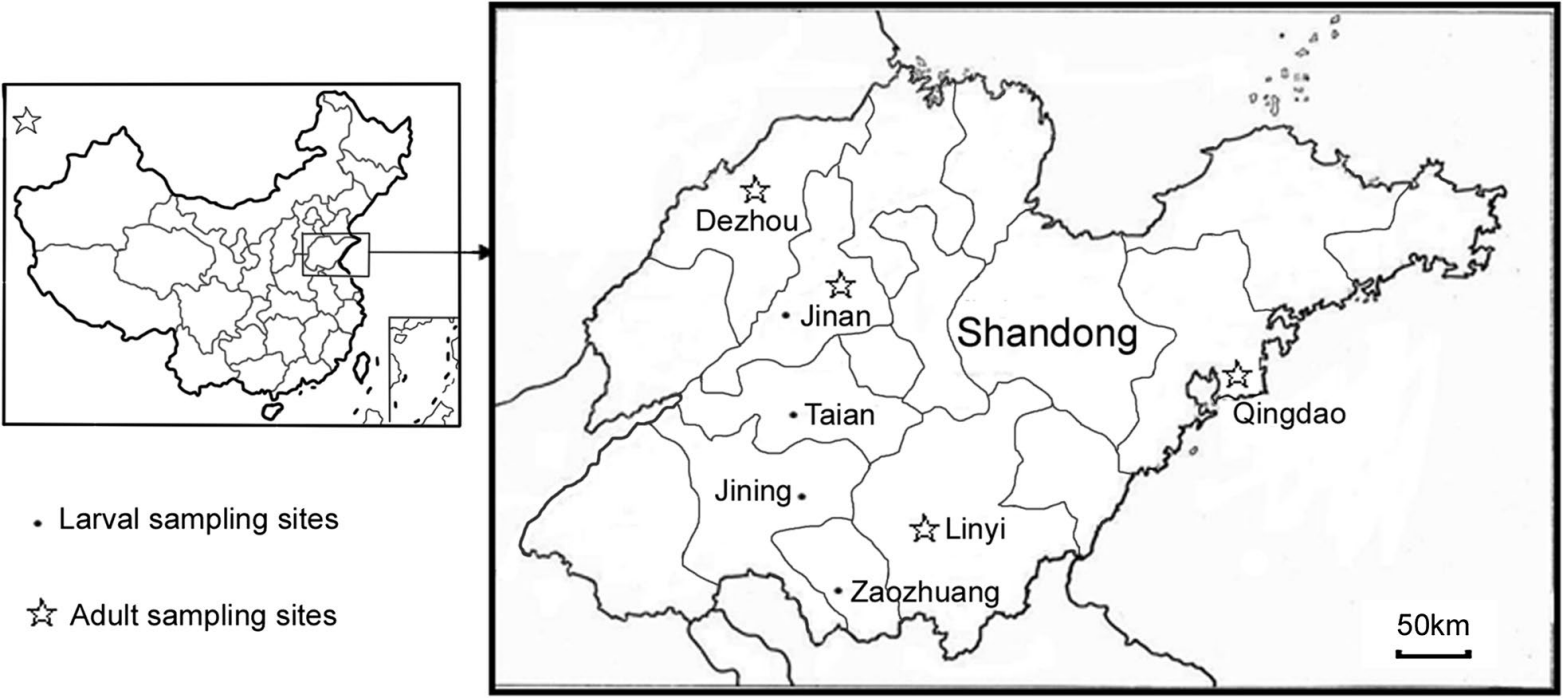
Map of mosquito sample sites in Shandong Province, China

This study was conducted in Jining, Zaozhuang, Jinan, Taian, Linyi, Dezhou and Qingdao, in Shandong Province (Fig. 1, Additional file 1: Table S1), a coastal area in east China with a warm temperate monsoon climate and concentrated rainfall during the summer season. Larvae were collected from Jining in 1992, 1994, 2001, 2004, 2011, 2014 and 2018; Zaozhuang in 1993, 1995, 2001, 2004, 2011, 2014 and 2018; Jinan in 1994, 1995, 2004, 2011, 2014 and 2018; Taian in 2001, 2011, 2014 and 2018; and Linyi, Dezhou and Qingdao in 2014 (Additional file 1: Table S1). The predominant agricultural activities in the area are wheat, rice and corn farming. The average annual rainfall for this region in the past 30 years was 750 mm, with 60–70% of rainfall concentrated in the months of June, July and August. The mosquito larval habitats in the study area included ponds, drains (collections of rainwater and effluents from factories and houses), rice fields and other stagnant aquatic habitats. All collection was done on public land. Due to severe insect pest damage to agriculture, insecticide use for pest control has been intensive in this region, with several rounds of spraying conducted in a single growing season. Pyrethroids have been commonly used for agricultural pest control since the 1980s [24] and additional insecticides, such as organophosphates and carbamates, have been employed in the study area since the 1990s.

### Larval sampling and preparation

Larvae were collected in Jining, Zaozhuang, Jinan, Taian, Linyi, Dezhou and Qingdao from the 1990s to 2018, with between 4 and 6 samples collected between over 10 and 26 years at each site. At each collection time, more than 800 larvae or pupae were collected from 20 local breeding sites (irrigated rice fields and drains on roadsides or collection sites of rainwater and effluents from factories and houses) using standard 350 ml dippers. The larvae were reared to adulthood before they were morphologically identified as *Cx. p. pallens*. All adults were fed a 10% sucrose solution. Females were blood-fed with defibrinated sheep blood using a Hemotek unit (Discovery Workshops, Accrington, United Kingdom). The blood-fed mosquitoes were transferred to a mosquito cage and allowed to lay eggs. The eggs were hatched, and larvae were reared in purified water with Tetramin fish food (Tetra, Melle, Germany). Larvae from the F1 generation from Jining, Zaozhuang, Jinan and Taian were reared to the third-instar and then used for the larval bioassays. For the adult insecticide resistance bioassay, 100–120 three- to five-day-old female mosquitoes were randomly selected from the F1 generation from Jinan, Linyi, Dezhou and Qingdao. Insecticide susceptible strains (protected from contact with insecticides for 20 years) that are routinely reared in our laboratory were employed as a reference.

### Larval insecticide resistance bioassay

The susceptibility of the third-stage larvae of the collected *Cx. p. pallens* was tested using five insecticides: cypermethrin, propoxur, deltamethrin, acetofenate and DDVP, with testing following the WHO guidelines [25]. Third-instar larvae (*n* = 20–25) were exposed to the pesticides in plastic vials with 100 ml of distilled water. Seven test concentrations were used: cypermethrin, 2–32 µg/l; propoxur, 0.28–1.35 mg/l; deltamethrin, 2.5–40 µg/l; acetofenate, 2.5–40 µg/l; and DDVP, 0.25–4 mg/l. After 24 h, the number of alive and dead larvae were recorded, and the LC_50_ was estimated with a log-probit model [26]. The test was repeated three times. The susceptible strains were exposed in plastic cups with distilled water or insecticide as a control before testing the wild population each time. Field-collected larvae were exposed to distilled water as a control, and if the mortality rate was greater than 20%, the experiment was invalidated. The resistance ratio (RR) was calculated as the ratio of the wild-strain LC_50_/susceptible-strain LC_50_.

### Adult insecticide resistance bioassay

Female adults from the 2014 collections were tested for susceptibility to deltamethrin using the standard WHO tube bioassay [5]. For each sample collection site, including Dezhou, Jinan, Linyi and Qingdao, in Shandong Province (Fig. 1), 100–140 female mosquitoes were exposed to WHO 0.05% deltamethrin insecticide-impregnated papers (the School of Biological Sciences, Universiti Sains Malaysia, Penang, Malaysia), with 20 to 25 mosquitoes per tube. For each sample collection, 5–7 replicates were performed. Carrier oil-treated papers without insecticide were obtained from the same source and used as a control. The mosquitoes were exposed for 1 h before transfer to recovery cups and were then maintained on a 10% sucrose solution for 24 h. The number of alive and dead mosquitoes was recorded.

### Molecular identification and detection of *kdr* mutations

One leg of each mosquito was employed for DNA extraction with the SYBR Green Extract-N-Amp Tissue PCR Kit (Sigma, Missouri, USA). Briefly, the mosquito leg was placed at the bottom of a 500 μl Eppendorf tube. Then, 100 μl of extraction solution and 25 μl of tissue preparation solution were added, followed by incubation at room temperature for 10 min, then for 10 min at 95 °C. After incubation, 100 μl of neutralization solution B was added, and the sample was mixed *via* vortexing. The extracted DNA was stored at 4 °C or used immediately for PCR. Molecular identification of *Cx. p. pallens* species was conducted using species-specific primers targeting amplification of the DIIS6 (domain 2 S6) region of the gDNA sequence of the *Cx. p. pallens para*-sodium channel gene α subunit (GenBank accession number, BN001092). To identify point mutations of the *Vgsc* gene at position 1014, we amplified a 521 bp fragment using the primer pair [27]: *kdr*-F 5′-CCT GCC ACG GTG GAA CTT C-3′ and *kdr*-R 5′-GGA CAA AAG CAA GGC TAA GAA-3′. The PCR products were directly sequenced using the Big-Dye kit of Sangon Biotech Co., Ltd. (Shanghai, China). A total of 217 *Cx. p. pallens* mosquitoes, ranging between 36–47 individuals per population, were subjected to this initial *kdr* genotyping.

To establish the association between *kdr* mutations and phenotypic resistance, 471 female adults identified as deltamethrin resistant or deltamethrin susceptible were screened from four sampling sites using the standard WHO tube bioassay [5]. In the present study, a resistant individual was defined as a mosquito that was still alive after the 24 h recovery period, and a susceptible mosquito was defined as a mosquito that was dead after the 24 h recovery period. This screen yielded a total of 173 resistant and 298 susceptible mosquitoes from Dezhou, Jinan, Linyi and Qingdao. The phenotyped mosquitoes were genotyped for *kdr* mutations at the 1014 codon *via* direct sequencing.

### Statistical analysis

The mortality rate of the larvae or adult mosquitoes exposed to insecticide was adjusted by the mortality rate of the mosquitoes in the control group (exposed to still water in larval bioassays or carrier oil-treated papers in adult bioassays), according to Abbott’s formula. For the *kdr* survey in multiple populations, mutation frequencies at the 1014 codon were calculated for each population. For non-synonymous mutations, a Hardy-Weinberg equilibrium test was performed using Fisher’s exact test with Bonferroni corrections to determine the heterozygote deficit in each population. For association between *kdr* mutations and resistance, Fisher’s exact test was performed and the odds ratio was determined for each *kdr* allele. Statistical analyses of differences in the larval insecticide resistance results were conducted with one-way ANOVA followed by LSD tests (homogeneity of variance: *P* > 0.05) or Dunnett’s T3 tests (homogeneity of variance: *P* < 0.05). Multivariate binary regression analysis was conducted to determine the correlation coefficient between the RR value and sampling time. Binary regression analysis was used to test associations among resistance phenotype, genotypes and populations. Statistical analyses were carried out using the SPSS software (version 19 for Windows, SPSS Inc., Chicago, USA)

The larval insecticide resistance level was evaluated by using the RR as reported by Keiding [28], Lai [29] and the WHO [30]: susceptibility (RR < 3); decreased susceptibility (RR = 3–5); low resistance (RR = 5–10); moderate resistance (RR = 10–40); high resistance (RR = 40–160); and very high resistance (RR > 160). According to the WHO [5], resistance evaluation indicators, mortality rates for the susceptible (S), suspected resistant (SR), and resistant (R) groups were identified as between 98–100%, 90–98% and < 90%, respectively.

## Results

### Larval insecticide resistance bioassay

**Fig. 2:**
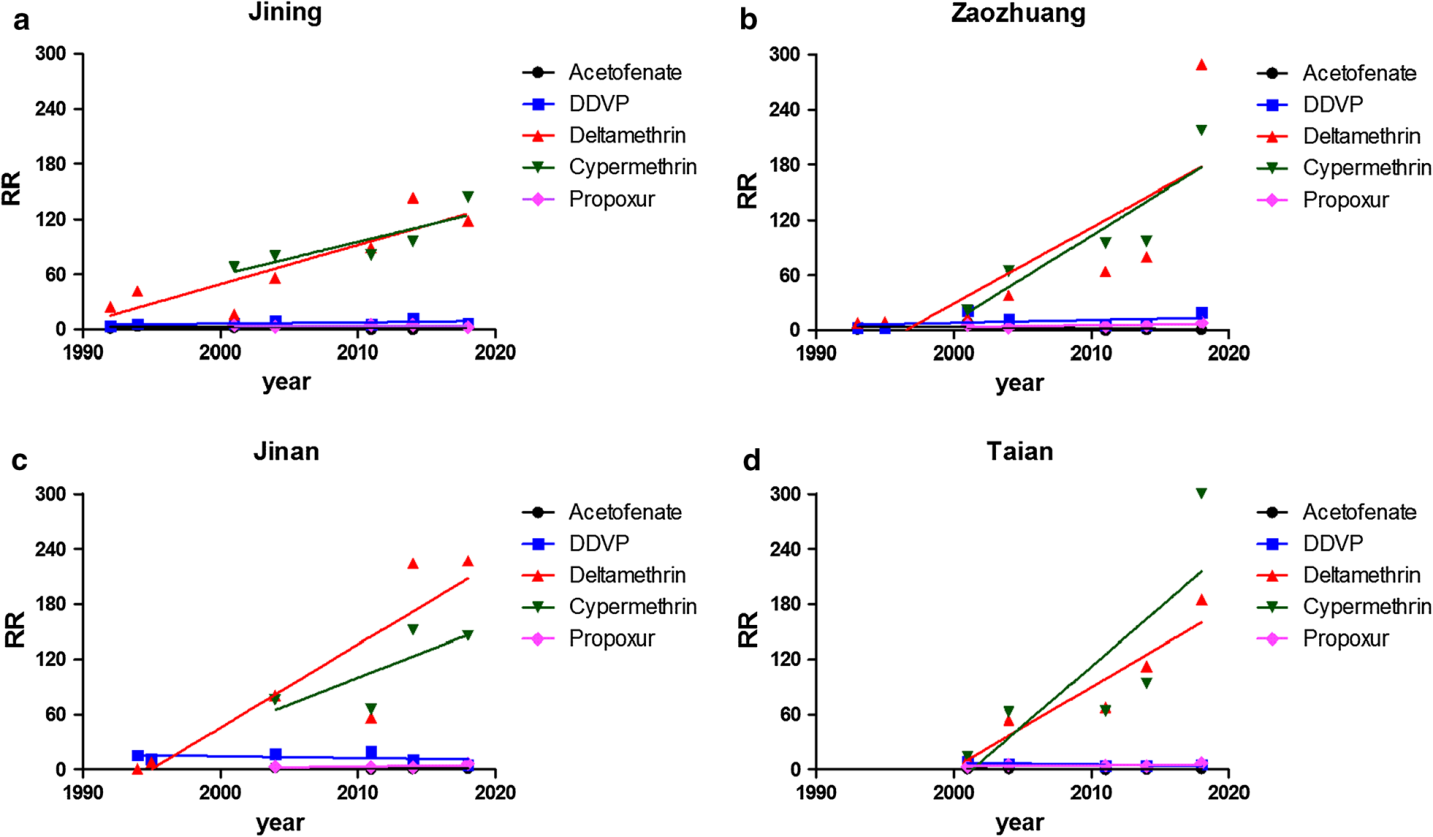
WHO standard insecticide resistance larvae bioassay from four *Culex pipiens pallens* populations in Shandong, China. The resistance ratio (RR) was calculated as the ratio of the wild-strain LC_50_/susceptible-strain LC_50_

The insecticide susceptibility bioassay data showed that the resistance ratio varied with location and year. Based on the WHO criteria, the *Cx. p. pallens* populations from Jining, Zaozhuang, Jinan and Taian showed increased resistance against deltamethrin and cypermethrin in the 2010s compared with low-level or mid-level resistance in the early 1990s (Fig. 2, Additional file 2: Table S2). Mosquito populations showed a range from susceptible to low resistance (RR_acetofenate_ = 0.05–8.01, RR_propoxur_ = 2.37–8.10) to acetofenate and propoxur, and a range from decreased susceptibility to moderate resistance against DDVP (RR_DDVP_ = 2.22–20.13). Levene’s test indicated heterogeneity of variance between groups in all samples (all *P* < 0.05). Welch’s (unequal variance) ANOVA was then conducted to compare means between groups (Jining: *F*_(4,11.937)_ = 14.982, *P* < 0.001; Zaozhuang: *F*_(4,11.798)_ = 3.934, *P* = 0.029; Jinan: *F*_(4,8.197)_ = 12.788, *P* = 0.001; Taian: *F*_(4,8.640)_ = 15.732, *P* = 0.001) and RR values for deltamethrin and cypermethrin were much higher than those for the other tested insecticides by Dunnett’s T3 test (Additional file 3: Table S3).

In order to determine the correlation coefficient between the RR value and sampling time, multivariate binary regression analysis was conducted. Resistance against deltamethrin showed a continuous increasing trend from the 1990s until 2018, and the difference was statistically significant (Table 1, Fig. 2). Mosquito resistance against the other insecticides (DDVP, propoxur and acetofenate) remained at a consistently low level (Table 1, Fig. 2).

**Table 1:** Multivariate regression analysis results for the effect of sampling time on resistance ratio (RR) in larval bioassays of *Culex pipiens pallens* populations. RR was calculated as the ratio of the wild-strain LC_50_/susceptible-strain LC_50_

City	Insecticide	Equation	*R* ^2^	*F*	*P*
Jining	Acetofenate	y = -0.0525x + 107.7	0.056	0.296	0.609
	DDVP	y = 0.1458x - 285.3	0.264	1.789	0.239
	Deltamethrin	y = 4.269x - 8488	0.771	16.803	0.009*
	Cypermethrin	y = 3.634x - 7209	0.726	7.956	0.067
	Propoxur	y = 0.02285x - 49.89	0.018	0.0536	0.832
Zaozhuang	Acetofenate	y = -0.1283x + 260.0	0.194	1.206	0.322
	DDVP	y = 0.3005x - 593.0	0.133	0.766	0.422
	Deltamethrin	y = 8.260x - 16490	0.621	8.205	0.035*
	Cypermethrin	y = 9.306x - 18600	0.807	12.541	0.038*
	Propoxur	y = 0.1675x - 331.3	0.434	2.297	0.227
Jinan	Acetofenate	y = -0.0574x + 116.8	0.214	0.545	0.537
	DDVP	y = 0.1919x - 398.4	0.135	0.622	0.475
	Deltamethrin	y = 9.037x - 18030	0.786	14.721	0.019*
	Cypermethrin	y = 5.838x - 11630	0.583	2.795	0.236
	Propoxur	y = 0.1781x - 354.6	0.366	1.154	0.395
Taian	Acetofenate	y = -0.02741x + 55.89	0.16	0.571	0.505
	DDVP	y = -0.2056x + 418.9	0.605	4.591	0.122
	Deltamethrin	y = 8.808x - 17610	0.885	23.047	0.017*
	Cypermethrin	y = 12.94x - 25890	0.661	5.860	0.094
	Propoxur	y = 0.1037x - 203.7	0.318	1.396	0.322

*P < 0.05

### Deltamethrin adult bioassays and *kdr* allele frequency distribution

A total of 217 *Cx. p. pallens* mosquitoes, ranging between 36–47 individuals per population, were subjected to *kdr* genotyping. Three alleles (wild-type L1014, L1014F and L1014S) and five genotypes were detected, indicating two mutations at codon 1014 (Table 2). At this codon, a change from the wild-type codon TTA (Leu) to TTT (Phe) or TCA (Ser) was detected at all four sampled sites, with the frequency ranging between 0–0.080 for the L1014S mutation and 0–0.386 for the L1014F mutation. Genotypic frequencies of field populations showed a significant departure from Hardy-Weinberg equilibrium (*P* *<* 0.05) caused by a heterozygote deficit [31]. Moreover, we did not detect any individuals with a TTT/TCA genotype.

**Table 2:** The *kdr* genotype and allele frequency of phenotypes at codon 1014 determined in *Culex pipiens pallens* populations from Shandong Province

Site	*n*	L1014 genotype						Allele frequencies (%)			*P* (HWE)^a^
		TTA/TTA	TTT/TTT	TCA/TCA	TTA/TTT	TTA/TCA	TTT/TCA	L1014	1014S	1014F	
								TTA (%)	TCA (%)	TTT (%)	
Dezhou	47	22	12	1	10	2	0	0.596	0.043	0.362	<0.001
Jinan	44	17	13	1	8	5	0	0.534	0.080	0.386	<0.0001
Linyi	46	29	8	0	6	3	0	0.728	0.033	0.239	<0.0001
Qingdao	36	19	8	0	8	1	0	0.653	0.014	0.333	<0.05
Laboratory strain	44	44	0	0	0	0	0	1.000	0.000	0.000	–

^a^*P* refers to the *P-*value for the Chi-square test

*Abbreviation*: HWE, Hardy-Weinberg equilibrium

The mortality rates of *Cx. p. pallens* female mosquitoes following the 24 h recovery period after exposure to deltamethrin-impregnated paper are listed in Table 3. The samples from Dezhou, Jinan, Linyi and Qingdao were included in the R group, and those from the laboratory strains were included in the S group. During our survey period, the 1014 codon of the *Vgsc* gene was genotyped in a total of 471 female mosquitoes from the four Chinese *Culex* mosquito populations that were phenotyped for resistance or susceptibility to deltamethrin. Among these females, 173 individuals were classified as resistant (R) (alive after the 24 h recovery period in the WHO tube bioassay) and 298 were susceptible (S).

**Table 3:** WHO standard deltamethrin resistance adult bioassay results in *Culex pipiens pallens*

Sampling site	No. of specimens	Mortality (%) ± SE	Phenotype
Dezhou	100	52.17 ± 4.08	R
Jinan	120	39.04 ± 9.56	R
Linyi	120	70.68 ± 6.66	R
Qingdao	140	55.23 ± 5.53	R
Lab strain	115	98.96 ± 2.08	S

*Abbreviations*: R, resistant; S, susceptible; SE, standard error

To determine the impact of the *kdr* mutation at the 1014 codon on pyrethroid resistance, the L1014S and L1014F alleles were analyzed separately for their odds ratio associated with deltamethrin resistance. We found that the frequency of the L1014F mutation was significantly higher in the R population than that in the S population for all four populations (odds ratios ranging between 1.88–4.46, all *P* < 0.01) (Table 4). However, the L1014S mutation was not significantly associated with deltamethrin resistance in any of the populations (*P* > 0.05). In order to test associations among resistance phenotype, genotypes and populations, binary logistic regression analysis was performed, and it was found that resistance phenotype was associated with genotypes of *Cx. p. pallens* (*P* = 0.019), but not with different cities (*P* = 0.062). LL and FL genotypes were related to resistance phenotype, but LS and SS genotypes were unrelated to resistance phenotype, with FF as a reference category (Table 5).

**Table 4:** Association between *kdr* mutation and phenotypic resistance in *Culex pipiens pallens* from Shandong Province

Population	Phenotype	*n*	Genotype					Odds ratio (95% CI)		*P* ^a^	
			LL	LS	SS	FL	FF	L1014S	L1014F	L1014S	L1014F
Dezhou	S	47	26	4	0	10	7	1.68 (0.46–6.20)	2.63 (1.38–5.04)	0.32	<0.005
	R	43	15	2	2	8	16				
Jinan	S	38	21	5	1	7	4	0.83 (0.30–2.33)	1.88 (1.44–2.45)	0.46	<0.0001
	R	58	13	5	2	20	18				
Linyi	S	67	50	2	0	10	5	6.47 (1.23–34.42)	4.46 (2.16–9.21)	0.03	<0.0001
	R	28	10	3	1	4	10				
Qingdao	S	54	37	1	1	7	8	1.67 (0.36–7.65)	2.91 (1.53–5.53)	0.70	<0.0001
	R	44	18	2	1	10	13				

^a^Fisher’s exact probability test

*Abbreviations:* R, resistant; S, susceptible; LL, homozygous leucine/leucine; LS, heterozygotes leucine/serine; SS, homozygous serine/serine; FL, heterozygotes phenylalanine/leucine; FF, homozygous phenylalanine/phenylalanine; CI, confidence interval

**Table 5:** Logistic regression analysis showing the key factors

	B	*df*	*P*	Exp (B)
Cities	-0.180	1	0.062	0.835
Genotypes		4	0.019*	
Genotype (LL)	-0.947	1	0.001*	0.388
Genotype (LS)	-0.644	1	0.184	0.525
Genotype (SS)	0.321	1	0.715	1.379
Genotype (FL)	-0.757	1	0.036*	0.469
Constant	1.041	1	0.004	2.833

*Notes*: FF genotype is the reference category. Resistance phenotype is set to 1 and sensitive phenotype is set to 0. B is the regression coefficient. Exp (B): odds ratio

*P < 0.05

## Discussion

The present study is by far the most comprehensive survey of *Cx. p. pallens* insecticide resistance to be conducted in Shandong Province, China. Three important findings arose from this study. First, we conducted surveillance of five different insecticides from four insecticide categories over two decades, and the increasing resistance levels observed for deltamethrin indicated continuous elevation of insecticide selection pressure in all study areas. Secondly, an adult bioassay against pyrethroid insecticides conducted in 2014 indicated a high level of resistance. Thirdly, the L1014F *kdr* mutation of the *Vgsc* gene was positively related to mosquito survivorship to deltamethrin.

The use of chemical agents as prevention and control measures for mosquitoes is common practice due to the fact that some pesticides have high efficacy and low mammalian toxicity. Some organochlorine pesticides, with the exception of acetofenate, have been banned due to their toxicity and because of new insecticide development in the 1983 in China [8, 32]. Acetofenate was once a widely applied insecticide for both agriculture and public health purposes. In addition to this work, consistent resistance levels against acetofenate were detected in *Cx. p. pallens* in Hebei and Liaoning [33, 34] provinces in 2001 and 2004. In the most recent decade, *Cx. p. pallens* showed an RR against acetofenate of less than three, thus the mosquito population was sensitive to acetofenate. The resistance level against propoxur, a carbamate pesticide, remained steady and low in all four populations; similarly, *Cx. p. pallens* have been found to be susceptible in Hebei Province [35]. DDVP is the most frequently used organophosphate pesticide in households, and monitoring has shown a low resistance level against DDVP in Tianjin and Liaoning provinces [2]. In recent years, pyrethroids (especially deltamethrin and cypermethrin) have become the most widely used pesticides, due to their high efficiency and low toxicity. These insecticides are extensively employed as IRS or incense or used to impregnate bednets [7]. Pyrethroid resistance increased significantly from the 1990s to 2018, with resistance against deltamethrin in the Zaozhuang population of 290-fold being observed in 2018 compared with 8-fold in 1993, and resistance against cypermethrin in the Taian population of 300-fold being observed in 2018 compared with 14-fold in 2001. Notably, only 17% of the geographical strains of *Cx. p. pallens* from 12 provinces in China were found to be highly resistant to deltamethrin from 1997 to 2002 [34], while mosquitoes from Beijing, Hebei and Hainan provinces have all developed high resistance to deltamethrin in adult bioassays in recent years [36]. A similar situation was found in Thailand, where the insecticide resistance of *Cx. p. pallens* has increased from sensitive to high resistance [15]. The intensive use of pyrethroids has triggered severe insecticide resistance in this vector and resulted in reduced efficacy of vector control in China [2]. Therefore, the determination of insecticide resistance and clarification of the mechanism by which it has developed are of great importance.

Using mosquito samples collected from four sites in 2014, we established that adult female *Cx. p. pallens* showed strong resistance against deltamethrin. This result is consistent with the high larval resistance levels ranging from RR = 40 in Linyi to RR = 224 in Jinan. Pyrethroids are primarily used for personal protection in domestic applications in urban environments [37, 38]. Ultra-low-volume sprays and long-term use of pyrethroids in surrounding agricultural fields may also have accelerated selection for pyrethroid resistance.

High resistance against pyrethroids is consistent with the prevalence of the L1014F and L1014S *kdr* mutations in the tested *Cx. p. pallens* populations. A modest frequency of *kdr* mutations was observed in the four resistant populations compared with susceptible populations. This modest *kdr* mutation frequency may be related to intensive pyrethroid usage in the past two decades in an area where major campaigns were carried out to eradicate mosquitoes, in addition to the wide adoption of pyrethroids for spraying economic crops [3, 16, 39–41]. Pyrethroids have been the major insecticide employed for aerial spraying for adult mosquito control and agriculture pest control. Therefore, monitoring the *kdr* mutation frequency may be a useful surveillance method for monitoring pyrethroid resistance in *Cx. p. pallens*.

The L1014F mutation was found to be positively correlated with the deltamethrin-resistant phenotype, as reported previously in *Cx. p. pallens* [42], *Cx. quinquefasciatus* [43] and *An. gambiae* [44]. Subsequently, the additional mutation at this same codon (L1014S) was reported, but has only appeared in some *Cx. p. pallens* populations from Japan [45], China [42] and *Cx. pipiens* complex from the USA [46]. In this study, the homozygotes for both the L1014F and L1014S mutations were detected in all four populations, but the TTT/TCA mutation was not observed in any individual mosquitoes. Only the L1014F mutation, and not L1014S, appears to confer protection against deltamethrin in *Cx. p. pallens* populations, with odds ratios ranging between 1.88–4.46 at the four sampling sites. The role of the L1014F mutation in insecticide resistance has been validated by the demonstration that this mutation reduced channel sensitivity to pyrethroids [15, 27]. In a previous study of *Cx. p. pallens* the L1014F mutation was found to correlate significantly with deltamethrin resistance and respond to deltamethrin selection in laboratory selection experiment [42]. The occurrence of the L1014S mutation was not associated with deltamethrin resistance in our study, which was consistent with other research [47]. Shi et al. [47] exposed a field-collected population of *Cx. p. pallens* to deltamethrin of LC_50_ concentration for multiple generations and found that the frequency of resistant allele L1014F increased progressively up to 100% at generation 14, while the frequency of LI014 and L1014S reduced. However, the L1014S mutation in *Cx. pipiens* has been shown to be associated with a low level of resistance to pyrethroids, but much more strongly with resistance to DDT [48].

In the present study, only third-instar larvae were used to examine resistance levels against the insecticides. In a previous study [49], *Cx. p. pallens* larvae showed and increasing trend in resistance from instar I to instar IV. In different developmental stages, P450 enzymes and other metabolic enzymes occur at different levels inside the larval body, and metabolic activities therefore vary at different stages. Thus, it is recommended that insecticides be applied in early larval stages, when larvae are very sensitive to chemicals, to prevent or delay resistance development.

We recognize several limitations of our study. First, among the six collection times, we only carried out bioassay adult mosquitoes for pyrethroid resistance in 2014, and other insecticide categories were not included in this analysis. Secondly, is the use of larval assays for chemicals used as adulticides. Due to logistical constraints, it was not possible for us to also collect the large number of larvae required for conducting adult resistance bioassays in every year. Thirdly, a survey of the *kdr* mutation frequency over more years would be informative. Fourthly, we did not examine combined metabolic enzyme activities to test metabolic levels in the mosquitoes in the present study, which may contribute the strong resistance phenotypes observed.

## Conclusions

The findings of this work have important implications for *Cx. p. pallens* control. First, there is an urgent need for the development of a surveillance plan for pyrethroid resistance and countermeasures to control the spread of resistance, as a wide distribution of the *kdr* mutations was found in *Cx. p. pallens* mosquitoes. It is possible that *Cx. p. pallens* populations in the field may still be susceptible to pyrethroids. Secondly, the significant positive association between *kdr* mutation and mosquito survivorship suggests that *kdr* mutations might be a viable biomarker for surveying pyrethroid resistance in *Cx. p. pallens*. Although the potential role of metabolic detoxification enzymes and other resistance mechanisms in pyrethroid resistance calls for further studies, we emphasize that more research is needed to validate the correlation between *kdr* mutations and pyrethroid resistance at the population level. The rapid increase in insecticide resistance and the wide distribution of *kdr* mutations in *Cx. p. pallens* mosquitoes call for the development of a resistance surveillance plan and for the management of insecticide efficacy.

## Additional files

**Additional file 1: Table S1.** Mosquito sampling sites and times.

**Additional file 2: Table S2.** WHO standard insecticide resistance larvae bioassay from five *Culex pipiens pallens* populations in Shandong Province.

**Additional file 3: Table S3.** Multiple comparisons by Dunnett’s T3 test.

## Abbreviations

Vgsc: voltage-gated sodium channel
kdr: knockdown resistance
WNV: West Nile virus
ITNs: insecticide-treated bednets
IRS: indoor residual spray
DDVP: dichlorvos
IIS6: domain II of subunit 6
RR: resistance ratio
